# Effects of Cilostazol and Aspirin on Diabetic Foot Ulcer and Peripheral Artery Disease: A Retrospective Study

**DOI:** 10.7759/cureus.80929

**Published:** 2025-03-20

**Authors:** J.A. Jayalal, Selwyn Kumar, Abhinand Mohan

**Affiliations:** 1 General Surgery, Kanyakumari Government Medical College, Asaripallam, IND

**Keywords:** aspirin, cilostazol, dfu, fontaine classification, peripheral arterial diseases

## Abstract

Diabetic foot ulcer (DFU) remains a serious complication of diabetes, with a significant prevalence of peripheral artery disease (PAD) in affected patients. PAD complicates DFU healing, reducing recovery rates significantly. In other settings, cilostazol, a phosphodiesterase-3 inhibitor, has shown promise in reducing the risk of arterial thrombosis more effectively than aspirin, despite the traditional recommendation of aspirin to mitigate the heightened cardiovascular risk associated with diabetes. This study retrospectively compared the effects of cilostazol and aspirin on wound healing and PAD symptoms in patients with DFU. We evaluated 60 patients with DFU and PAD treated with either cilostazol or aspirin retrospectively for wound healing progression, clinical improvement, and alleviation of PAD symptoms. As 10 patients’ data were incomplete, it included 50 patients from two cohorts.* *There were 30 patients in the cilostazol cohort and 20 in the aspirin cohort. We assessed the wound using Wagner's classification and planimetric techniques and classified the PAD stages using the Fontaine classification. Results indicated that cilostazol treatment led to a significantly higher rate of complete wound healing (90%) compared to aspirin (55%) and a faster healing timeline. Cilostazol also demonstrated a more effective improvement in PAD symptoms, facilitating a better quality of life for patients. These findings suggest that cilostazol may offer a more effective treatment option for promoting wound healing and managing PAD in DFU patients than aspirin. We recommend further randomized and controlled studies to validate these results and refine DFU treatment protocols. Protocols that incorporate cilostazol could lead to significant advancements in patient care, ultimately reducing the burden of diabetic foot ulcers. As healthcare providers seek more effective therapies, understanding the mechanisms behind cilostazol's efficacy will be crucial for optimizing treatment strategies.

## Introduction

One of the most dreaded consequences of diabetes mellitus is diabetic foot ulcer (DFU). According to studies, persons with type 1 diabetes have a 25% lifetime risk of getting DFU [1]. In addition, over 50% of patients with DFU have peripheral artery disease (PAD) [2,3]. According to studies, infections and PAD have a significant impact on how well DFU patients recuperate. This study examined DFU patients with and without PAD, finding that individuals without PAD recovered at a rate of 84%, while patients with PAD recovered at a rate of 69%. Because diabetic patients have a high risk of developing cardiovascular disease, studies advise using aspirin in modest doses. Studies have noted a higher risk of thrombosis in individuals with diabetes mellitus who have coronary stents [4,5]. Individuals with diabetes mellitus also face an increased risk of platelet failure [5]. Studies have shown that cilostazol is more effective than aspirin in lowering the risk of arterial thrombosis [6,7]. However, potential side effects and contraindications, particularly in patients with certain heart conditions, prevent the universal prescription of cilostazol [8]. Consequently, healthcare providers must carefully weigh the benefits and risks when considering antiplatelet therapy for diabetic patients with a history of cardiovascular issues. This study compared the effects of cilostazol and aspirin on wound healing in patients with DFU and PAD.

## Materials and methods

The study retrospectively analyzed data from patients with diabetic foot ulcers (DFUs) and peripheral artery disease (PAD) treated in the General Surgery department. Patients were categorized into two cohorts: one receiving cilostazol and the other receiving aspirin. Data were collected on demographic parameters, comorbidities, wound characteristics, treatment modalities, and clinical outcomes. The primary outcome measures included wound healing progression, assessed using Wagner’s classification and planimetric techniques, and the severity of PAD, graded using the Fontaine classification.

The inclusion criteria for this study were diabetic foot ulcers classified as Wagner grades 1, 2, 3, or 4, with documented peripheral arterial disease, and patients between the ages of 18 and 70 years. Patients were excluded if they had a history of malignancy, were immunocompromised, had non-diabetic foot wounds due to vascular or dermatological conditions, suffered from nephropathy, or did not have PAD. Patients were divided into two treatment cohorts: the cilostazol cohort, which received 100 mg of cilostazol once daily for the first week, followed by an increased dose of 100 mg twice daily, and the aspirin cohort, which received 100 mg of aspirin once daily. The average treatment duration for both groups was three months.

To assess wound healing, a clinical examination was performed to evaluate the presence of infection, necrosis, ischemia, and ulcer size. Wound depth was categorized using Wagner-Meggitt’s classification. Additionally, planimetric techniques were used to measure the wound surface area. The presence of infection was determined based on bacterial culture results, with antibiotic therapy adjusted according to culture sensitivity findings. Patients suspected of osteomyelitis underwent radiographic evaluation using X-ray, CT, or MRI as needed.

Peripheral arterial disease severity was assessed using the Fontaine classification, which categorizes PAD based on symptoms ranging from asymptomatic disease to critical limb ischemia. Clinical evaluation included the presence of claudication, rest pain, and non-palpable pedal pulses. Doppler ultrasound was used to assess arterial blood flow, and the ankle-brachial index (ABI) was measured to quantify vascular insufficiency. An ABI of less than 0.9 was considered indicative of PAD, while values below 0.3 were classified as critical limb ischemia. In patients with chronic kidney disease, the toe-brachial index was measured instead. Neuropathy was assessed using monofilament and vibration tests. The monofilament test was performed using a 5.07 Semmes-Weinstein filament applied to eight distinct areas on the sole, while vibration perception was evaluated using a tuning fork applied to the malleolus and great toe. Wound healing response to treatment was classified based on the percentage of granulation tissue formation [9]: no response: <25% of granulation tissue, minimal response: 25-50% of granulation tissue, partial response: 51-75% of granulation tissue, complete response: > 75% of granulation tissue.

Statistical analysis

The statistical analysis was conducted using IBM Corp. Released 2024. IBM SPSS Statistics for Windows, Version 30.0. Armonk, NY: IBM Corp. Continuous variables were expressed as mean ± standard deviation (SD) or median with interquartile range, depending on data distribution. Categorical variables were presented as frequencies and percentages. The normality of continuous variables was assessed using the Shapiro-Wilk test. Comparisons between the cilostazol and aspirin groups were performed using the independent t-test for normally distributed continuous variables and the Mann-Whitney U test for non-normally distributed variables. Categorical variables were analyzed using the chi-square test or Fisher’s exact test where appropriate. A p-value of less than 0.05 was considered statistically significant.

## Results

The retrospective data were collected for 60 patients, and as there was noncompliance with data criteria in 10 patients' records, they were excluded, and a total of 50 patients were included in this study. The 50 patients were divided into two cohorts: 30 patients received cilostazol, and 20 patients received aspirin therapy. The cilostazol cohort had 24 (80%) males and six (20%) females, with a median age of 55.5 ± 10.2 years. Associated comorbidities noted were chronic obstructive pulmonary disease (COPD) in two patients (6.66%) and hypertension (HT) in 16 (53.3%). The mean duration of the disease was 18.4 years (1-30 years), and the duration of DFU diagnosis was 2.4 months (1-8 months). Eight (26.6%) of the patients had previously undergone amputations; of these, seven (23.3%) had Wagner's grade 2, 16 (53.3%) had grade 3, and seven (23.3%) had grade 4 DFU. Twelve (40.0%) of the patients had osteomyelitis, with a mean ABI index of 0.90. The aspirin cohort had 20 patients; of those, four (20%) were female and 16 (80%) were male, with the median age being 54.8 ± 9.2 years. Associated comorbidities noted were chronic obstructive pulmonary disease (COPD) in two patients (10%) and hypertension (HT) in eight (40%). The mean duration of the disease was 16.8 years (9-29 years), and the duration of DFU diagnosis was 2.2 months (1-7 months). Five (25%) of the patients had previously undergone amputations; of these, five (23) had Wagner's grade 2, 11 (55) had grade 3, and four (20) had grade 4 DFU. Seven (35.0%) of the patients had osteomyelitis, with a mean ABI index of 0.90. There was no statistically significant difference. However, in the recovery and duration of treatment, there was a statistically significant advantage in the cilostazol cohorts. In the cilostazol group, there were 27 patients (90%) who had a full recovery, compared to 11 patients (55%) in the aspirin group (p < 0.05). The other patients showed partial response. In the cilostazol group, the mean treatment time was 1.31 months (1-2 months), but in the aspirin group, it was 1.82 months (1-2.5 months) (p < 0.05).

The data on demographical and clinical features are shown in Table 1.

**Table 1:** Demographical and baseline characteristics and clinical outcome of the patients.

Demographical and clinical features	Cilostazol cohort (n. 30)	Aspirin cohort (n-20)	P-value
Mean age ± SD in years	55.5 ±10.2	55.8±9.2	0.65
Gender distribution (%) (%)			
Females	6 (20)	4 (20)	0.79
Males	24 (83.3)	16 (80)	0.64
Duration of diabetes years/median (25th to 75th percentile)	18.4 (10-30)	16.8 (9-39)	0.55
Duration of diabetic foot ulcer in months	2.4 (1-8)	2.2 (1-7)	0.87
Neuropathy (%)	23 (76)	16 (78)	0.76
Infection (%)	24 (80)	12 (60)	0.40
Necrosis (%)	15 (50)	5 (25)	0.26
Wound size (cm) median (25th 75th percentile)	8.4 (2-25)	7.6 (5-25)	0.42
Amputation stump (%)	9 (30)	5 (25)	0.89
Osteomyelitis (%)	12 (40)	7 (35)	0.46
Mean duration of treatment in months (25th 75th percentile)	1.31 (1-2)	1.82 (1-2.5)	<0.05
Wagner-Meggitt’s classification, n (%)			
Grade 1	0 (0)	0 (0)	
Grade 2	7 (23.3)	5 (25)	0.55
Grade 3	12(40)	11 (55)	0.58
Grade 4	7 (23.3)	4 (20)	0.61
Outcome, n (%)			
Complete response (granulation tissue>75%)	17 (56.6)	8 (40)	<0.05
Partial response (granulation tissue 51-75%)	3 (10)	9 (45)	<0.05
No response	0 (0)	0 (0)	
Closing with graft (%)	6 (20)	11 (55)	<0.05
Complete close with skin (%)	10 (33.3)	3 (15)	<0.05

The vascularity of the limbs was assessed using physical, Doppler, and ankle-brachial indexes, and there were no statistically significant differences between the cohorts in terms of blood flow and perfusion. Furthermore, these findings suggest that both groups exhibited comparable vascular health, indicating that the interventions applied did not markedly influence the vascular parameters measured. Table 2 shows the findings.

**Table 2:** Peripheral arterial findings of patients.

Peripheral artery disease findings of patients	Cilostazol group (n. 30)	Aspirin group (n. 20)	P-value
Fontaine classification, n (%)			
Stage 1	1 0	0 (0)	
Stage 2A	6 (20)	9 (45)	0.26
Stage 2B	12 (40)	8 (40)	0.85
Stage 3	9 (30)	3 (15)	0.24
Stage 4	3 (10)	0 (0)	0.06
Doppler USG, n (%)			
Monophasic flow	6 (20)	4 (20)	0.15
Biphasic flow	19 (63.3)	10 (50)	0.66
Triphasic flow	5 (23.3)	6 (35)	0.52
Ankle Brachial Index (ABI) n (%)			
<0.95	22 (73.3)	13 (65)	0.34
0.95-1.30	6 (20)	7 (35)	0.48
>1.30	2 (6.6)	0 (0)	0.07

## Discussion

Patients with DFU were assessed for PAD in the research. As antiaggregant medications, clostazol and aspirin were utilized to compare the effects on wound healing. The literature contained an acceptable number of publications contrasting the effects of cilostazol and aspirin on wound healing. Despite the advantages of this study, some of its disadvantages include its retrospective nature and small patient population. Patients with DFU also have peripheral polyneuropathy, PAD, infection, and other comorbidities [3]. Nearly all DFU patients have diabetic neuropathy, and for the vast majority of them, ischemia is present in addition to the neuropathy [7]. In our study, 76% of patients in the cilostazol group and 78% of individuals in the aspirin group had peripheral neuropathy.

The first complaint with PAD, which is typically in the lower extremities, is discomfort that manifests as claudication [7]. Aches that don't go away with rest replace the pain in the later stages of the condition [7] due to the ischemia of the distal nerves. According to the Fontaine classification, all the patients in this study had stage 2A, 2B, 3, and 4 symptoms. Following treatment, the patients' leg symptoms improved in the cilostazol group by 86% and in the aspirin group by 65%. ABI 0.9, monophasic flow in the Doppler, claudication, resting discomfort, and non-palpable foot pulse are some of the techniques used to diagnose PAD 4 [10]. Ankle-brachial index (ABI) is a noninvasive test that measures the ratio of systolic pressure in the upper and lower extremities. However, ABI can be unreliable in people with diabetes depending on the degree of calcification in the peripheral arteries [11]. In these conditions, the ABI readings may exceed 1.3. For diabetic patients, because of the elevated cardiac risk, it is crucial even though it is not a reliable indication of vascular occlusion [11,12]. ABI>1.3 was discovered in two patients who were in the cilostazol group.

The present study compared the demographic, clinical, and therapeutic outcomes of two cohorts of patients with diabetic foot ulcers (DFUs) treated with either cilostazol or aspirin. The results highlight significant differences in treatment outcomes, providing valuable insights into the management of DFUs, a challenging complication of diabetes associated with high morbidity and healthcare costs. The two cohorts were well-matched in terms of demographic and clinical features, including mean age, gender distribution, duration of diabetes, duration of DFUs, and the prevalence of neuropathy, infection, necrosis, and osteomyelitis. This similarity in baseline characteristics ensures that the observed differences in outcomes are likely attributable to the treatment interventions rather than confounding variables. The absence of significant differences in Wagner-Meggitt’s classification further supports the comparability of the two groups at baseline.

The cilostazol cohort demonstrated significantly higher rates of complete response (56.6% vs. 40%, p < 0.05) and complete skin closure (33.3% vs. 15%, p < 0.05) compared to the aspirin cohort. This suggests that cilostazol may enhance wound healing by promoting granulation tissue formation and epithelialization. Cilostazol, a phosphodiesterase-3 inhibitor, is known to improve microcirculation and reduce platelet aggregation, which may contribute to its superior efficacy in DFU management. In contrast, the aspirin cohort had a significantly higher rate of partial response (45% vs. 10%, p < 0.05) and required more frequent grafting for wound closure (55% vs. 20%, p < 0.05). This indicates that while aspirin may facilitate some degree of wound healing, it is less effective than cilostazol in achieving complete resolution. Aspirin’s antiplatelet and anti-inflammatory properties may contribute to partial healing but may not be sufficient to address the complex pathophysiology of DFUs, which often involves impaired angiogenesis and chronic inflammation.

The aspirin cohort had a significantly longer mean duration of treatment (1.82 months vs. 1.31 months, p < 0.05). This prolonged treatment duration may reflect the slower healing process in this group, further underscoring the relative advantage of cilostazol in accelerating wound healing. The differential outcomes observed between the two cohorts can be attributed to the distinct mechanisms of action of cilostazol and aspirin. Cilostazol’s ability to improve blood flow by vasodilation and its antiplatelet effects may enhance tissue perfusion and oxygen delivery, critical factors in wound healing. Additionally, cilostazol has been shown to inhibit smooth muscle cell proliferation and reduce inflammation, which may further promote healing in chronic wounds like DFUs. On the other hand, while aspirin’s antiplatelet and anti-inflammatory properties are beneficial, they may not sufficiently address the microvascular and cellular dysfunctions inherent in DFUs. The findings of this study suggest that cilostazol may be a more effective therapeutic option for DFUs, particularly in achieving complete wound healing and reducing the need for surgical interventions such as grafting. However, aspirin may still have a role in cases where partial healing is acceptable or when grafting is planned as part of the treatment strategy. The choice of therapy should be individualized based on patient characteristics, wound severity, and treatment goals.

The distribution of PAD severity, assessed using the Fontaine classification, indicates that Stage 2B was equally prevalent in both groups (cilostazol: 40%, aspirin: 40%, p=0.85). Notably, the cilostazol group had a higher percentage of patients in Stage 3 (30% vs. 15%, p=0.24) and Stage 4 (10% vs. 0%, p=0.06), suggesting a possible trend toward more severe disease at baseline. While the differences did not reach statistical significance, these findings may reflect the preferential use of cilostazol in patients with more advanced claudication symptoms. Doppler ultrasound (USG) findings demonstrated monophasic flow in 20% of patients in both groups, indicating significant arterial insufficiency (p=0.15). Biphasic flow, which suggests moderate disease, was observed in 63.3% of the cilostazol group compared to 50% in the aspirin group (p=0.66). Conversely, triphasic flow, indicative of normal or near-normal arterial function, was more common in the aspirin group (35% vs. 23.3%, p=0.52). These findings suggest that while both groups had significant arterial impairment, the cilostazol group may have had relatively poorer baseline arterial function. ABI serves as a crucial diagnostic marker in PAD. In this study, a greater proportion of patients in the cilostazol group had an ABI of <0.95 (73.3% vs. 65%, p=0.34), indicating more significant arterial disease. Additionally, 6.6% of patients in the cilostazol group had an ABI of >1.30, whereas none in the aspirin group exhibited this finding (p=0.07). An ABI of >1.30 is often associated with arterial calcification and increased vascular stiffness, which may suggest differences in vascular compliance between the two treatment groups.

Cilostazol, a phosphodiesterase-3 inhibitor with vasodilatory and antiplatelet properties, is primarily indicated for the treatment of intermittent claudication in PAD. The observed trends toward more severe disease in the cilostazol group may reflect its preferential use in patients with disabling symptoms. Aspirin, a well-established antiplatelet agent, remains a cornerstone of PAD management, particularly for cardiovascular event prevention. The findings of this study highlight the need for individualized therapeutic approaches based on disease severity, hemodynamic parameters, and symptom burden. While this study provides valuable insights into the characteristics of PAD patients on cilostazol versus aspirin, certain limitations must be acknowledged. The sample size is relatively small, which limits the statistical power to detect significant differences. Additionally, the study lacks longitudinal follow-up to assess the impact of these therapies on functional outcomes and disease progression. Future research with larger, randomized trials is warranted to determine the optimal treatment strategy for different PAD subgroups.

In this study, we evaluated the wounds of diabetic foot ulcer patients utilizing Wagner's Megitt classification. Clinical practitioners employ many categories for diabetic foot ulcers (DFUs). The University of Texas classification assesses the lesion based on its depth, the presence of infection, and ischemia [13]. The SAD classification system categorizes ulcers according to their dimensions, surface area, depth, presence of sepsis, arteriopathy, and denervation [14]. The PEDIS classification method categorizes wounds based on perfusion, surface area, depth, infection, and sensation [15]. The American Infectious Diseases Council classifies diabetic foot injuries into three categories: mild, moderate, and severe. The Wagner-Meritt classification system, which classifies diabetic foot ulcers according to wound depth and gangrene severity, is easily understood and used in this study [13,16].

Patients with DFU are started on an antiplatelet or antithrombotic therapy during and after wound care, as diabetes mellitus increases the risk of PAD development, increases mortality, and increases the likelihood of amputations [17,18]. Diabetic foot lesions are accentuated by PAD atherosclerosis. It is well established that diabetic patients experience earlier onset, faster progression, and more aggressive atherosclerosis than other patients [7] and that DFU patients with PAD have a poorer likelihood of healing with treatment [4]. Because of this, treating PAD is crucial to treating infection-related illnesses like DFU. The flexibility of peripheral arteries and tissue perfusion is significantly reduced by medial sclerosis, which is characterized by calcification of the tunica media. Diabetes mellitus increases the risk of PAD development, which increases mortality and increases the likelihood of amputations [17]. PAD atherosclerosis accentuates diabetic foot lesions. Diabetic patients are known to experience earlier onset, faster progression, and more aggressive atherosclerosis than other patients [8], and DFU patients with PAD are less likely to heal with treatment [4]. Because of this, treating PAD is crucial to treating infection-related illnesses like DFU. Calcification of the tunica media, a hallmark of medial sclerosis, significantly reduces the flexibility of peripheral arteries and tissue perfusion.

Researchers have identified many microvascular issues in diabetic individuals, such as arteriovenous shunting and deteriorating vascular function [19]. Individuals with diabetes mellitus suffer from capillary hypoperfusion because of these adverse changes, which further impair wound healing [20,21]. Additionally, studies have linked functional issues with diabetic patients' platelets to an increase in cardiovascular risks [7,22]. According to one study, platelet surface flow was lower in diabetic patients than in non-diabetic individuals, and there were more platelet microparticles floating around in the bloodstream [23]. Therefore, it is necessary not only to combat DFU but also to combat PAD. Various papers [24-26] compare the effectiveness of aspirin and cilostazol in the treatment of PAD. The American Diabetes Association (ADA) advises aspirin to individuals with diabetes mellitus to lower their risk of cardiovascular disease since it permanently reduces the COX-1 enzyme's activity in platelets. Cilostazol specifically inhibits phosphodiesterase 3, leading to an increase in intracellular cAMP and active protein kinase. As a result, cilostazol performs vasodilation in addition to inhibiting platelet aggregation.

Studies comparing the two drugs in diabetic and non-diabetic patients have shown that iloprost is more efficient than aspirin. However, our examination of the literature did not find any trials contrasting aspirin with cilostazol in the treatment of diabetic patients' wounds. Because of the risk of PAD, all diabetic patients in our clinic receive either aspirin or cilostazol as part of their treatment. In this trial, patients in the cilostazol group exhibited higher rates of wound healing and complete closure with granulation tissue and skin compared to those in the aspirin cohort. Additionally, the cilostazol group's patients recovered from their leg problems more quickly.

## Conclusions

Starting an antiplatelet medication with aspirin or cilostazol is necessary for patients with DFU due to the risk of PAD. According to the findings of this study, cilostazol is more effective than aspirin at easing PAD symptoms, enabling a more comfortable lifestyle, and effectively treating DFU. Aspirin is less effective than cilostazol in promoting wound healing in DFU patients. However, additional research with randomized and controlled experiments is required. Effective treatment protocols should consider individual patient needs and potential side effects associated with each medication. Ultimately, a tailored approach that includes both pharmacological and non-pharmacological interventions may yield the best outcomes for patients suffering from diabetic foot ulcers.
